# Intermittent Hypoxia Inhibits Hepatic CYP1a2 Expression and Delays Aminophylline Metabolism

**DOI:** 10.1155/2022/2782702

**Published:** 2022-04-29

**Authors:** Xiao-Bin Zhang, Xiao-Yang Chen, Kam Yu Chiu, Xiu-Zhen He, Jian-Ming Wang, Hui-Qing Zeng, Yiming Zeng

**Affiliations:** ^1^Department of Pulmonary and Critical Care Medicine, Zhongshan Hospital, Xiamen University, School of Medicine, Xiamen University, Teaching Hospital of Fujian Medical University, Siming District, Xiamen, Fujian Province, China; ^2^Department of Pulmonary and Critical Care Medicine, the Second Affiliated Hospital of Fujian Medical University, Second Clinical Medical College of Fujian Medical University, Center of Respiratory Medicine of Fujian Province, China; ^3^Department of Pulmonary and Critical Care Medicine, Quanzhou First Affiliated Hospital of Fujian Medical University, Fuzhou, China

## Abstract

**Purpose:**

In this study, we aimed to determine the effects of intermittent hypoxia (IH) on hepatic cytochrome P450 1A2 (CYP1A2) expression and the pharmacokinetics of CYP1A2-mediated aminophylline and warfarin *in vitro* and in a rabbit model of obstructive sleep apnea.

**Materials:**

Human normal liver (LO-2) cells were exposed to 30 min each of 1%, 1–21%, 21%, and 21–1% O_2_, and then, CYP1A2 expression and drug concentrations were analyzed. We compared the pharmacokinetic parameters of drugs administered to normoxic rabbits and those exposed to 10 min of IH during which the oxygen level fluctuated from 21% to 8%–10% (*n* = 10 per group).

**Result:**

s. The expression of CYP1A2 protein *in vitro* was significantly reduced in the IH compared with the normoxic cells (0.56 ± 0.11 vs. 1.27 ± 0.17, *p* < 0.001). Aminophylline was more abundant in cell culture supernatants after 48 h of IH than in those under normoxia. The *T*_1/2_, AUC_0–24 h_, and Ke values for aminophylline were significantly higher in the IH group.

**Conclusion:**

Intermittent hypoxia inhibits hepatic CYP1A2 expression and delays aminophylline metabolism, suggesting that the impact of IH on the expression of CYP enzymes should be closely monitored in clinical practice.

## 1. Introduction

Hypoxia significantly influences the expression of hepatic cytochrome P450 (CYP) enzymes and drug metabolism. Numerous experimental and clinical studies have shown that acute, chronic, or high-altitude hypoxia can affect the metabolism of many CYP substrates and relevant drugs, extending the half-life of the drugs [1–5]. These findings indicate that doses of drugs targeting hepatic CYP enzymes should be adjusted under conditions of sustained intermittent hypoxia (IH). Only a few studies have evaluated the impact of intermittent hypoxia (IH), a hallmark of obstructive sleep apnea (OSA), on CYP expression and associated drug metabolism.

Obstructive sleep apnea is associated with liver diseases, and IH contributes to cellular damage in the liver [6]. Our previous electron microscopy findings revealed that chronic IH leads to hepatocellular edema and rough endoplasmic reticulum in liver cells. Decreased theophylline metabolism has been attributed to an IH-induced decrease in total CYP levels [7]. Chronic IH combined with isoniazid and rifampicin, two first-line antituberculosis drugs, causes ultrastructural damage to liver cells [8]. We also found decreased total hepatic CYP 450 expression in mice exposed to chronic IH. After 12 weeks of IH exposure, the mRNA and protein levels of CYP1A2, one of the most essential CYP enzymes, were downregulated whereas those of other CYP enzymes remained unchanged [9]. Whether such findings can be replicated *in vitro* and whether the pharmacokinetics of drugs metabolized by specific CYP enzymes are altered awaits elucidation.

We aimed to verify our previous findings in a large animal model and evaluate the effects of IH on the pharmacokinetics of drugs that specifically require CYP1A2 expression and function for metabolism.

## 2. Materials and Methods

### 2.1. Reagents

We aimed to determine the influence of IH on CYP expression and drug pharmacokinetics in normal human liver cells. Therefore, we used LO-2 cells (Cell Center of the Shanghai Institutes for Biological Sciences, Chinese Academy of Sciences, Shanghai, China), which are considered a human normal liver cell line according to previous studies on CYP expression and drug cytotoxicity [10, 11]. Twenty adult male New Zealand white rabbits weighing 2.0–2.5 kg were provided by the Animal Center of Fujian Medical University. Cell Counting Kit-8 (CCK-8) was purchased from Beyotime (Beijing, China). An FITC-labeled Annexin V (Annexin V-FITC) apoptosis detection kit was obtained from BD Biosciences (China). TRIzol reagent was purchased from Invitrogen (Carlsbad, CA, USA). The PrimeScript^TM^ RT reagent Kit and SYBR Green I were purchased from Takara Biotechnology (Dalian, China). Aminophylline and warfarin were obtained from Sangon Biotech (Shanghai, China). High-performance liquid chromatography reagents were obtained from Thermo Fisher Scientific (San Jose, CA, USA).

### 2.2. Cell Culture, IH Exposure, and Drug Administration

**Figure 1:**
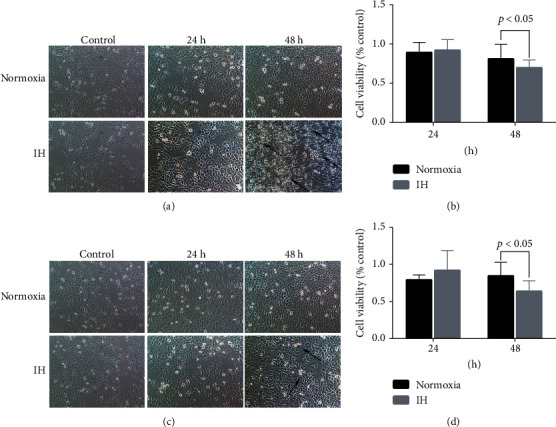
Effects of IH, aminophylline, and warfarin on liver cell viability. IH exposure for 48 h decreased viability (OD450 value) of cells incubated with aminophylline (a) and (b) and warfarin (c) and (d). IH, intermittent hypoxia; OD, optical density.

LO-2 cells were cultured in Dulbecco's modified Eagle medium containing 10% fetal bovine serum at 37°C under a 5% CO_2_ atmosphere. The cells were divided into normoxia and IH groups (*n* = 6 dishes per group, of which three in each group contained aminophylline and warfarin). The IH group was exposed to IH in an oxygen control incubator (Smartor 118, Guangzhou, China) under the following conditions: 30 min each at 1%, 1%–21%, 21%, and 21%–1% O_2_ for 48 h [12]. Normoxic cells were maintained under a 5% CO_2_ atmosphere throughout the study. We calculated the 50% inhibitory concentration (IC_50_) for aminophylline and warfarin to determine the appropriate nontoxic concentrations required to incubate the cells (Figure 1). Based on the IC values, we incubated the cells with 500 *μ*mol/L of each drug for 48 h to synchronize with IH. All experiments were repeated in triplicate.

### 2.3. Evaluation of Cell Viability

Cells (1 × 10^4^/well) were seeded in 96-well plates at baseline. Furthermore, viability after 24 and 48 h of IH was determined using the CCK-8 assay following the manufacturer's instructions. The absorbance of each well was measured at a 450 nm wavelength.

### 2.4. Flow Cytometry

LO-2 cells were collected, washed with cold phosphate-buffered saline, resuspended in 1 × binding buffer, and then stained with Annexin V-FITC and propidium iodide at the indicated time points. Ratios (%) of apoptotic cells were determined by flow cytometry using a BD FACSCalibur^TM^ (BD Biosciences) as described by the manufacturer.

### 2.5. Quantitative Reverse Transcription Polymerase Chain Reaction (RT-qPCR)

Total RNA was isolated from LO-2 cells, reverse-transcribed into cDNA, and then sequences of interest were amplified by qPCR as described [9] using the following forward and reverse (5′ ⟶ 3′) primers: CYP1A2 : ACCCAGCTTCCTCATCCTCC and CCATCAGCTCCTGCAACCTG; hypoxia-inducible factor (HIF)-1*α*: AGTTCCGCAAGCCCTGAAAGC and GCAGTGGTAGTGGTGGCATTAGC; GAPDH : ATCAGCAATGCCTCCTGCAC and ACAGTCTTCTGGGTGGCAGT. The temperature cycling program comprised one step at 95°C for 30 s, followed by 40 cycles at 95 for 30 s and 60 for 30 s. The internal control was glyceraldehyde-3- phosphate dehydrogenase (GAPDH). Relative expression levels were calculated using the 2^−ΔΔCT^ method. All samples were analyzed in triplicate.

### 2.6. Animals, IH Exposure, and Drug Administration

**Figure 2:**
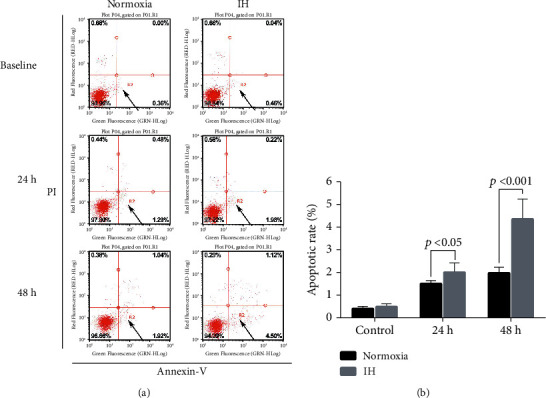
Effects of IH on liver cell apoptosis. Apoptosis rates were significantly higher in IH than in normoxic liver cells at 24 and 48 h (*p* < 0.05 and *p* < 0.001, respectively). IH, intermittent hypoxia; PI, propidium iodide.

Animal experiments complied with the Guide for the Care and Use of Laboratory Animals, Institute for Laboratory Animals, issued by the National Institute of Health [13]. The rabbits were acclimatized in cages for 1 week with free access to food and water. The rabbits were randomly assigned to normoxia and IH groups (*n* = 10 each). Two or three rabbits were exposed to IH (10 min cycles of oxygen fluctuating from 21% to 8%–10%, 8 h/d, for 5 consecutive weeks [14]) in a plexiglass chamber (120 × 125 × 80 cm^3^) as described with some modifications (Figure 2) [9, 14]. All rabbits were fasted overnight prior to drug administration. Five rabbits in each group were injected with 4 mg/kg aminophylline in 4 mL of 5% glucose [15, 16] in an ear vein at 8 : 00 AM on the last day of IH. The other five rabbits in each group received 0.2 mg/kg warfarin in 5 mL saline [17, 18] by oral gavage on the first day of the last week of IH for pharmacokinetic studies.

### 2.7. Plasma Extraction and Liver Sample Preparation for Pharmacokinetic Studies

Blood (1 mL) was collected from the auricular veins of the rabbits at 0.17, 0.33, 0.5, 1.0, 2.0, 3.0, 4.0, 5.0, 6.0, 8.0, 12, and 24 h after aminophylline injection and at 0.5, 1.0, 1.5, 2, 4, 8, 12, 24, 48, 72, 96, 120, and 144 h after warfarin gavage. Plasma was isolated and stored at −80°C. After the last IH cycle, the rabbits were euthanized by injecting pentobarbital sodium (20 mg/kg) into the carotid artery. Whole livers were removed, weighed, and divided, and approximately 100 g of liver tissue was stored at −80°C.

### 2.8. Western Blotting

Protein levels in lysed LO-2 cells and homogenized liver cells were determined by western blotting as previously described [9]. Cell samples were blotted with 1 : 1,000-diluted rabbit anti-CYP1A2 and 1 : 5,000- diluted GAPDH polyclonal antibodies (Proteintech Group Inc., Rosemont, IL, USA), CYP2C9 polyclonal antibody (1 : 1, 000, Abcam, ab4236), rabbit anti-CYP2C19 polyclonal antibody (1 : 1, 000, Abcam, ab137015), rabbit anti-CYP3A4 polyclonal antibody (1 : 1, 000, Abcam, ab124921), and mouse anti-hypoxia induced factor-1*α* monoclonal antibody (1 : 1000, Novus Biologicals, Littleton, CO, USA). Liver samples were blotted with 1 : 1000-diluted goat anti-CYP1A1+CYP1A2 (ab4227) polyclonal and 1 : 5,000-diluted mouse anti-GAPDH (ab9482) monoclonal antibodies (Abcam).

### 2.9. Quantitation of Aminophylline and Warfarin

Drug concentrations were assessed by HPLC [19, 20] and DAS for CDM 2.0 software [21]. The area under the curve from 0 to *t* (AUC_0-36 or 144 h_) and 0 to ∞ (AUC_0-∞_), half-life (*T*_1/2_), elimination rate constant (K_e_), apparent volume of distribution (V_d_), clearance (CL), and peak maximum concentration (*C*_max_) were calculated.

### 2.10. Statistical Analysis

The data were statistically analyzed using GraphPad Prism 5.0 (GraphPad Software, Inc., San Diego, CA, USA) and SPSS 22.0 software (IBM Corp., Armonk, NY, USA). The normality of numerical variables was assessed, and the results are presented as means ± standard deviation (SD). If the data were normally distributed, significant differences between two independent and among multiple groups were compared using Student's *t*-tests and one- or two-way ANOVA, respectively. Values with *p* < 0.05 were considered statistically significant.

## 3. Results

### 3.1. Intermittent Hypoxia and Aminophylline Reduced Cell Viability

**Figure 3:**
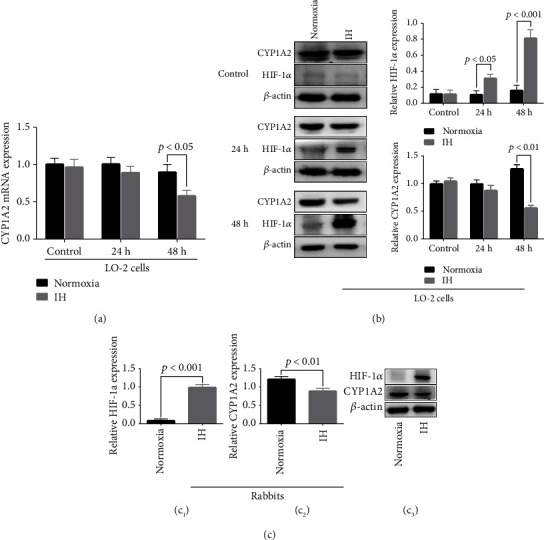
Effects of IH on CYP1A2 and HIF-1*α* expression in vitro and in vivo.

Figures 1 and 3 show significantly reduced LO-2 cell viability after 48 h of IH in the aminophylline and warfarin groups (*p* < 0.05). The decreased cell viability was caused by IH rather than aminophylline or warfarin.

### 3.2. Intermittent Hypoxia Increased Apoptosis

The flow cytometry results revealed a high apoptotic rate among LO-2 cells exposed to IH for 24 and 48 h (*p* < 0.05 and *p* < 0.001, respectively; Figure 2).

### 3.3. Intermittent Hypoxia Inhibited CYP1A2 and Increased HIF-1*α* Expression in Liver Cells In Vitro and In Vivo

Compared to the cultured LO-2 cells and liver samples from rabbits in the normoxia group, those from rabbits exposed to IH showed reduced CYP1A2 protein expression (*in vitro*: 0.56 ± 0.11 in the IH group vs. 1.27 ± 0.17 in the normoxia group, *p* < 0.001; *in vivo*: 0.89 ± 0.21 in the IH group vs. 1.22 ± 0.22 in the normoxia group, *p* < 0.01). CYP1A2 mRNA expression in LO-2 cells was also decreased in the IH group (0.58 ± 0.19 in the IH group vs. 0.92 ± 0.21 in the normoxia group, *p* < 0.05). HIF-1*α* protein expression was increased by IH both in *in vitro* and in liver samples from rabbits exposed to IH *in vivo* (0.56 ± 0.11 *vs.* 1.27 ± 0.17 *p* < 0.001*in vitro*; 0.89 ± 0.21 *vs.* 1.22 ± 0.22, *p* < 0.01*in vivo*). Compared with normoxia, IH similarly decreased the expression of CYP1A2 mRNA in LO-2 cells (0.58 ± 0.19 *vs.* 0.92 ± 0.21, *p* < 0.05). Compared with normoxia *in vitro* and *in vivo*, IH increased the expression of HIF-1*α* protein (0.32 ± 0.12 *vs.* 0.11 ± 0.11, *p* < 0.05 at 24 h *in vitro*; 0.8 ± 0.24 *vs.* 0.17 ± 0.15, *p* < 0.001*in vitro* at 48 h; 1.00 ± 0.24 *vs.* 0.09 ± 0.12, *p* < 0.001*in vivo*; Figure 3).

### 3.4. Intermittent Hypoxia Did Not Alter Other CYP Enzymes

**Figure 4:**
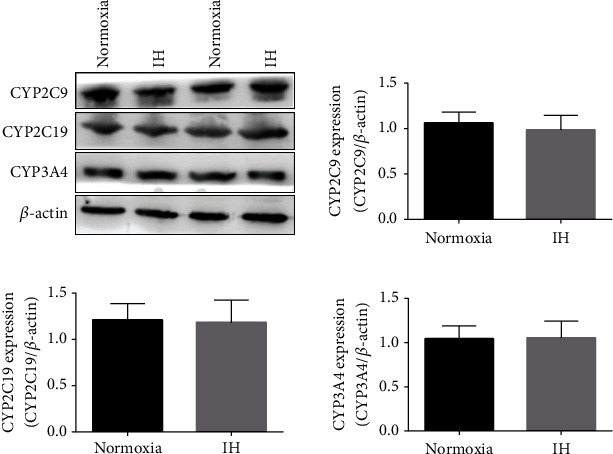
Influence of IH on expression of CYP enzymes responsible for warfarin metabolism. Western blots show no differences in CYP2C9, CYP2C19, and CYP3A4 expression between normoxic and IH groups (*p* < 0.05 for all). Expression of CYP enzymes is shown relative to that of *β*-actin.

Exposure to IH did not alter the CYP2C9, CYP2C19, and CYP3A4 enzymes responsible for warfarin metabolism (Figure 4).

### 3.5. Intermittent Hypoxia Inhibited Aminophylline Metabolism In Vitro

**Figure 5:**
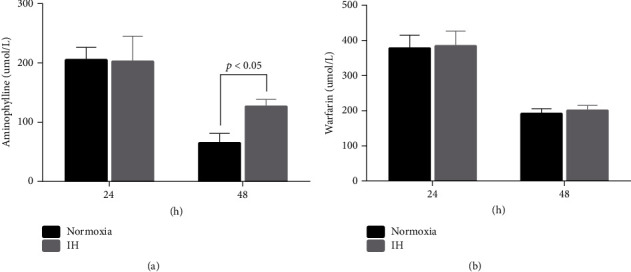
Effects of IH on aminophylline and warfarin concentrations in vitro. (A, B) Concentrations of aminophylline and warfarin did not differ between normoxic and IH LO-2 cells after 24 h, but that of aminophylline after 48 h was significantly higher in the IH cells than that in the normoxic cells (125.83 ± 12.84 vs. 64.33 ± 16.61 *μ*mol/L, *p* < 0.001). The warfarin concentration remained similar between the IH and normoxic cells at 48 h (190.50 ± 14.88 vs. 200.00 ± 14.63 *μ*mol/L, *p* < 0.05). IH, intermittent hypoxia.

The abundance of aminophylline in LO-2 cells was increased after an IH exposure for 48 h compared with that under normoxia (125.83 ± 12.84 *μ*mol/L *vs*. 64.33 ± 16.61 *μ*mol/L, *p* < 0.001) but not after 24 h (Figure 5(a)). In contrast, intracellular warfarin levels did not significantly differ after either 24 or 48 h exposure of IH (Figure 5(b)). Standard curves and chromatographs are shown in Figures 4 and 5.

### 3.6. Intermittent Hypoxia Inhibited Aminophylline but Not Warfarin Metabolism in Rabbit Models

**Figure 6:**
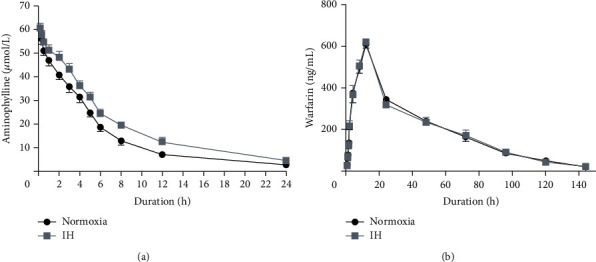
Mean plasma concentration-time curves for aminophylline and warfarin in the rabbit model of IH. (a) Time curves of mean plasma aminophylline concentrations in IH and normoxic rabbits intravenously injected with 4 mg/kg aminophylline. (b) Time curves of mean plasma warfarin concentrations in IH and normoxic rabbits orally administered with 0.2 mg/kg warfarin. IH, intermittent hypoxia.

The average weight over time and average live weight did not significantly differ between the normoxic and IH rabbits (Figures 6 and 7, respectively). The average plasma concentrations of the two drugs throughout the experiment are listed in Tables 1 and 2, and the average plasma concentration-time curves are shown in Figure 6. Tables 1 and 2 summarize the pharmacokinetic parameters for intravenously injected aminophylline and the orally administered warfarin. Values for the *T*_1/2_, K_e_, CL, AUC_0–24 h_, and AUC_0-∞_ of aminophylline significantly differed between the normoxia and IH groups (*p* < 0.001 for all) whereas those of warfarin did not (*p* < 0.05 for all).

## 4. Discussion

The present study showed that IH downregulated CYP1A2 expression in liver cells *in vitro* and *in vivo.* This subsequently influenced the concentration of aminophylline, requiring CYP1A2 catalysis or reduced aminophylline clearance in the rabbit models. However, IH did not influence the clearance of warfarin, which is metabolized by other CYPs.

Drug metabolism mediated by CYP enzymes is oxygen dependent. Hypoxia is one of the most important factors that modulate hepatic CYP enzyme expression. Hypoxia might interrupt the biotransformation of drugs metabolized in the liver. Chronic sustained high-altitude hypoxia significantly decreases the activity and expression of CYP3A1, suggesting downregulated CYP3A4 expression/activity, which could explain changes in the pharmacokinetics of lidocaine among native Tibetan and Han Chinese individuals [22]. Recent experimental findings [23, 24] are consistent with early reports that SH leads to the downregulation of CYP1A2 expression. These studies mainly investigated the effects of acute or chronic SH, rather than IH, on hepatic CYP expression and the functional metabolism of relevant drugs. Unlike SH, IH, which is a unique hallmark of OSA, contributes to systemic inflammation, endothelial injury, and oxidative stress [25]. The physiopathology of IH might resemble that of ischemia-reperfusion. An association between IH and hepatic damage has been indicated. Chronic IH leads to mild liver damage in lean mice *via* oxidative stress and excessive glycogen accumulation in hepatocytes [26]. We previously found ultrastructural changes such as hepatocellular edema and fuzzy rough endoplasmic reticulum in liver cells from mice exposed to IH [7, 8]. Levels of total cytochrome P450 [7], as well as CYP1A2 mRNA and protein expression [9], were also decreased in the livers of mouse models of IH. Our results were consistent with the findings in rat models of OSA [27].

The pharmacokinetics of drugs that require hepatic CYP enzymes for metabolism in patients or large rodent models of OSA or those exposed to IH have remained unknown. The present findings showed that IH increased HIF-1*α* expression and reduced CYP1A2 expression *in vitro* and *in vivo*. The aminophylline concentration in the supernatant of normal human liver cell cultures increased after IH exposure. Moreover, the *T*_1/2_ of aminophylline was prolonged, and the clearance rate was decreased in rabbits after 5 weeks of IH exposure. However, the pharmacokinetic parameters of warfarin, which are mostly transformed by the CYP2C superfamily [17, 28], were not affected by IH. We considered that the differential performance of the two drugs under IH could not be explained by either the IH-induced reduction in cell viability or the increase in apoptosis. The present and previous results indicate that aminophylline metabolism is catalyzed by CYP1A2 [15, 23, 29, 30], whereas warfarin is mostly transformed by the CYP2C superfamily [17, 28, 31], such as CYP2C9 and CYP2C19, rather than CYP1A2. This might partly explain why aminophylline was affected by IH whereas warfarin was not.

The mechanisms underlying changes in CYPs might be activated by serum inflammatory biomarkers caused by hypoxia or by a direct pre and/or posttranscriptional effect of hypoxia on hepatocytes [32]. Regarding the mechanism through which IH affects CYP expression levels, we previously revealed increased HIF-1*α* and nuclear factor-*κ*B (NF-*κ*B) expression in mice exposed to IH [9]. Intermittent hypoxia decreases the expression of glucocorticoid receptors in a rat model of sleep apnea [27]. In a rat model of IH and emphysema, the decreased expression of CYPs induced by IH is associated with increased expression of NF-*κ*B and decreased expression of nuclear pregnane *X*, constitutive androstane, and glucocorticoid receptors [33]. We postulate that IH leads to systemic inflammation and oxidative stress that further activates several signaling pathways such as HIF-1*α*/NF-*κ*B, which might contribute to the decreased CYP expression.

This study has several limitations. Intermittent hypoxia decreased cell viability and increased apoptosis rates, and to exclude the impact of these on drug metabolism is difficult. However, this concern was mitigated by the finding that only the concentration of aminophylline increased, whereas that of warfarin remained unchanged in cells exposed to IH *in vitro*. We administered only a single dose of aminophylline and warfarin to the rabbits, and we could not analyze the effects of IH on plasma drug concentrations and related pharmacokinetic parameters. Due to different pharmacokinetic parameters, the intravenous and oral administration of aminophylline and warfarin might affect the reliability of our results. Further studies using pharmacological tools or gene-targeting techniques to modulate CYP1A2 during IH *in vitro* or in animal models *in vivo* are warranted to establish a key link between changes in CYP1A2 expression and altered aminophylline metabolism.

## 5. Conclusion

The present findings showed that IH reduces the expression and activity of CYP1A2 and delays the metabolism of aminophylline, which is likely catalyzed by CYP1A2. Therefore, the dosage of drugs catalyzed by CYP1A2 might require adjustment in patients with OSA.

## Figures and Tables

**Table 1:** Effects of IH on pharmacokinetic parameters of aminophylline.

Parameters	Normoxia (*n* = 5)	IH (*n* = 5)
*T*_1/2_ (h)	3.89 ± 1.39	5.15 ± 2.26^*∗*^
K_e_ (L/h)	178.25 ± 6.20	134.75 ± 5.52^*∗*^
V_d_ (mL/kg)	64.75 ± 9.90	65.63 ± 3.02
CL (mL/h/kg)	12.0 ± 0.76	9.13 ± 0.64^*∗*^
AUC_0–24 h_ (mg/h/L)	289.06 ± 13.69	358.24 ± 16.00^*∗*^
AUC_0-∞_ (mg/h/L)	327.62 ± 18.30	454.40 ± 30.82^*∗*^

The data are shown as means ± SD. AUC_0-∞_, area under curve-time profile curve from time 0 to infinity; AUC_0-t_, area under curve-time profile curve from time 0 to end; K_e_, elimination rate constant; CL, clearance; *T*_1/2_, half-time; V_d_, apparent volume of distribution; ^*∗*^*p* < 0.05 compared with normoxic cells.

**Table 2:** Effects of IH on pharmacokinetic parameters of warfarin (mean ± SD, *n* = 10).

Parameters	Normoxia (*n* = 5)	IH (*n* = 5)
*C*_max_ (ng/L)	607.03 ± 16.20	618.80 ± 3.86
*T*_1/2_ (h)	28.10 ± 7.20	23.97 ± 2.63
AUC_0–144 h_ (mg/h/L)	27684.34 ± 1181.92	27358.69 ± 1339.80
AUC_0-∞_ (mg/h/L)	28820.98 ± 1837.82	28112.48 ± 1347.09
CL (L/h/kg)	0.001 ± 0.00	0.001 ± 0.00

The data are shown as means ± SD. AUC_0-∞_: area under curve-time profile curve from time 0 to infinity; AUC_0-t_: area under curve-time profile curve from time 0 to end. CL: clearance; *C*_max_: peak of maximum concentration; *T*_1/2_: half-life.

## Data Availability

All the data generated in this study are available in the main manuscript and in supplementary information.

## Abbreviations

AUC: Area under curve
CCK-8: Cell Counting Kit-8
CL: Clearance
Cmax: Maximum concentration
CYP: Cytochrome P450
HIF-1: Hypoxia induced factor-1
IC50: 50% inhibitory concentration
IH: Intermittent hypoxia
Ke: Elimination rate constant
NF-B: Nuclear factor-B
OSA: Obstructive sleep apnea
qRT-PCR: Quantitative real-time polymerase chain reaction
SH: Sustained hypoxia
T1/2: Half-time
Vd: Apparent volume of distribution.

## Supplementary Materials

Supplementary Materials Supplementary Materials. Table 1. Aminophylline concentrations (mg/L) at different time points (*n* = 10). Table 2. Warfarin concentrations (ng/mL) at different time-points (*n* = 10). Figure 1. IC50 values of aminophylline and warfarin. Figure 2. Oxygen ratios in a chamber containing rabbits. Figure 3. Effect of IH on cell viability. Figure 4. Standard curve and chromatographs of aminophylline. Figure 5. Standard curve and chromatographs of warfarin. Figure 6. Body weight of rabbits at different time points. Figure 7. Liver weight of normoxic and intermittently hypoxic rabbits. Western blot and RT-PCR analysis showed that the expression of CYP1A2 mRNA and protein was downregulated while that of HIF-1*α* protein was upregulated by IH exposure in vitro (both *p* < 0.001; (a) and (b) and in liver tissues of IH group rabbits compared with those of normoxic rabbits (c). Expression of CYP enzymes and HIF-1*α* is shown relative to that of *β*-actin. CYP, cytochrome P450; HIF, hypoxia induced factor; IH, intermittent hypoxia; RT-PCR, real-time polymerase chain reaction.
