# Supplementary material for: Intermittent Hypoxia Inhibits Hepatic CYP1a2 Expression and Delays Aminophylline Metabolism
Source: Evid Based Complement Alternat Med. 2022 Apr 29;2022:2782702. doi: 10.1155/2022/2782702 (PMC9076297; doi:10.1155/2022/2782702)
Supplement: Supplementary Materials — Table 1. Aminophylline concentrations (mg/L) at different time points (n = 10). Table 2. Warfarin concentrations (ng/mL) at different time-points (n = 10). Figure 1. IC50 values of aminophylline and warfarin. Figure 2. Oxygen ratios in a chamber containing rabbits. Figure 3. Effect of IH on cell viability. Figure 4. Standard curve and chromatographs of aminophylline. Figure 5. Standard curve and chromatographs of warfarin. Figure 6. Body weight of rabbits at different time points. Figure 7. Liver weight of normoxic and intermittently hypoxic rabbits. Western blot and RT-PCR analysis showed that the expression of CYP1A2 mRNA and protein was downregulated while that of HIF-1α protein was upregulated by IH exposure in vitro (both p < 0.001; (a) and (b) and in liver tissues of IH group rabbits compared with those of normoxic rabbits (c). Expression of CYP enzymes and HIF-1α is shown relative to that of β-actin. CYP, cytochrome P450; HIF, hypoxia induced factor; IH, intermittent hypoxia; RT-PCR, real-time polymerase chain reaction. [file 2782702.f1.docx]

Table 1 Aminophylline concentration (mg/L) at different time-point (n=10)

| Time(h) | Normoxia group | IH group |
| --- | --- | --- |
| 0.17 | 60.188±1.987 | 60.588±1.872 |
| 0.33 | 55.925±2.191 | 57.925±1.563**^*^** |
| 0.50 | 51.100±2.071 | 54.663±1.382**^*^** |
| 1.00 | 46.913±2.301 | 51.238±2.249**^*^** |
| 2.00 | 40.775±2.095 | 48.238±2.451**^*^** |
| 3.00 | 35.913±2.453 | 43.175±2.400**^*^** |
| 4.00 | 31.388±2.367 | 36.263±1.942**^*^** |
| 5.00 | 24.738±1.710 | 31.413±2.055**^*^** |
| 6.00 | 18.725±1.877 | 24.488±1.476**^*^** |
| 8.00 | 12.888±1.852 | 19.563±1.091**^*^** |
| 12.00 | 7.113±1.100 | 12.513±1.702**^*^** |
| 24.00 | 2.838±0.883 | 4.575±1.314**^*^** |

**^*^**：*p*<0.05 when compared with normoxia group.

eTable 2 Warfarin concentration (ng/mL) at different time-point (n=10)

| Time(h) | Normoxia group | IH group |
| --- | --- | --- |
| 0.5 | 31.463±9.632 | 25.600±8.469 |
| 1.0 | 72.988±19.687 | 68.213±17.140 |
| 1.5 | 135.963±28.976 | 125.000±16.443 |
| 2.0 | 216.853±15.839 | 216.450±26.063 |
| 4.0 | 373.438±43.411 | 367.638±42.285 |
| 8.0 | 501.638±30.523 | 504.038±28.758 |
| 12.0 | 607.025±16.202 | 618.800±13.855 |
| 24.0 | 342.700±23.097 | 317.675±12.335 |
| 48.0 | 238.638±11.790 | 233.513±24.376 |
| 72.0 | 162.125±19.368 | 170.988±25.807 |
| 96 | 86.813±13.341 | 90.913±6.311 |
| 120 | 49.250±13.584 | 43.538±5.065 |
| 144 | 21.125±5.070 | 21.688±2.356 |

**^*^**：*p*<0.05 when compared with normoxia group.

**eFigure 1 IC50 values of aminophylline and warfarin**


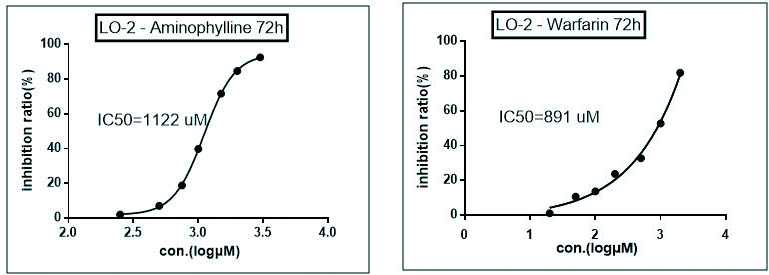


After 72 h culture, the IC50 values of aminophylline and warfarin to the cells were 1122 μmol/L and 891μmol/L.

**eFigure 2 Oxygen changes of the chamber adopting rabbits**





One hypoxia-oxygenation cycle consists of 10 minutes which maintaining the oxygen saturation in the chamber fluctuating from 21% to 8-10%. The S-450 oxygen detection alarm (IST-AIM Company, USA) with a sensitivity of 0.1% was used for the detection of chamber oxygen concentration.

**eFigure 3 Effect of IH on cell viability**


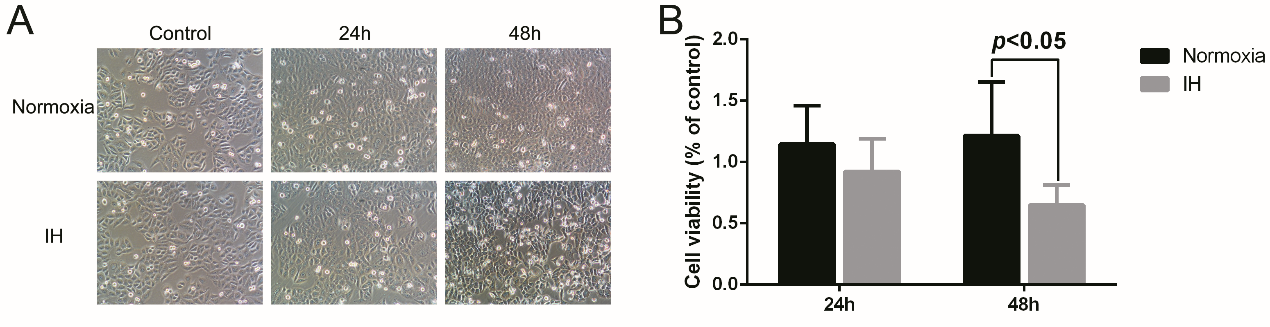


When compared with normoxia group, the cell viability in 24 h IH exposure group did not change, however, it markedly decreased in 48h IH exposure group.

IH: Intermittent hypoxia; OD: optical density.

**eFigure 4 Standard curve and chromatographs of aminophylline**


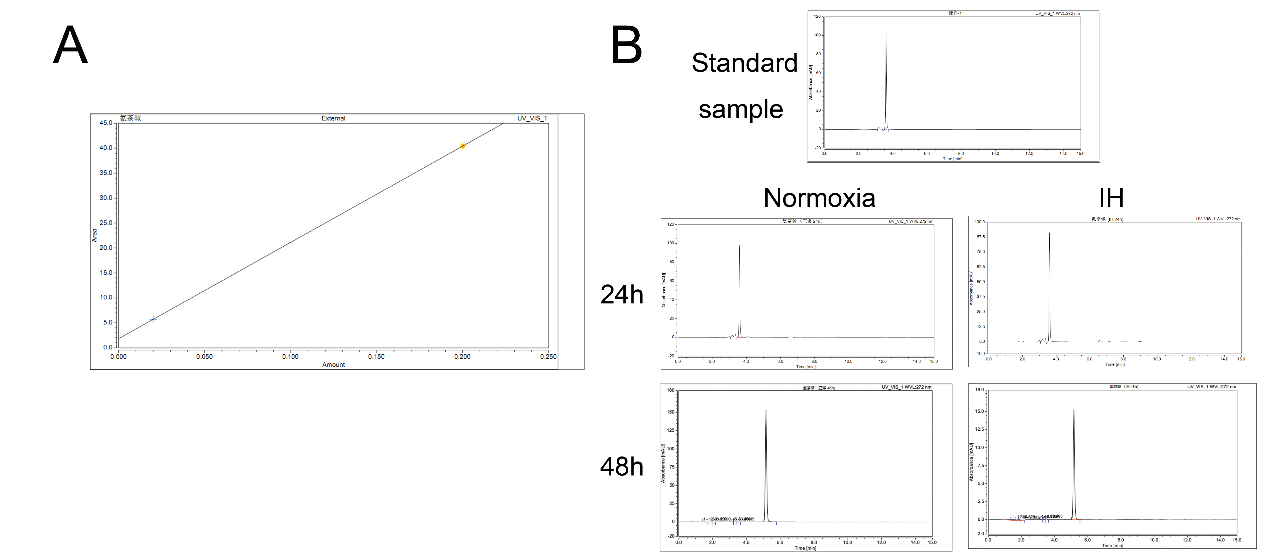


**A**: standard curve; **B**: chromatographs.

**eFigure 5 Standard curve and chromatographs of warfarin**


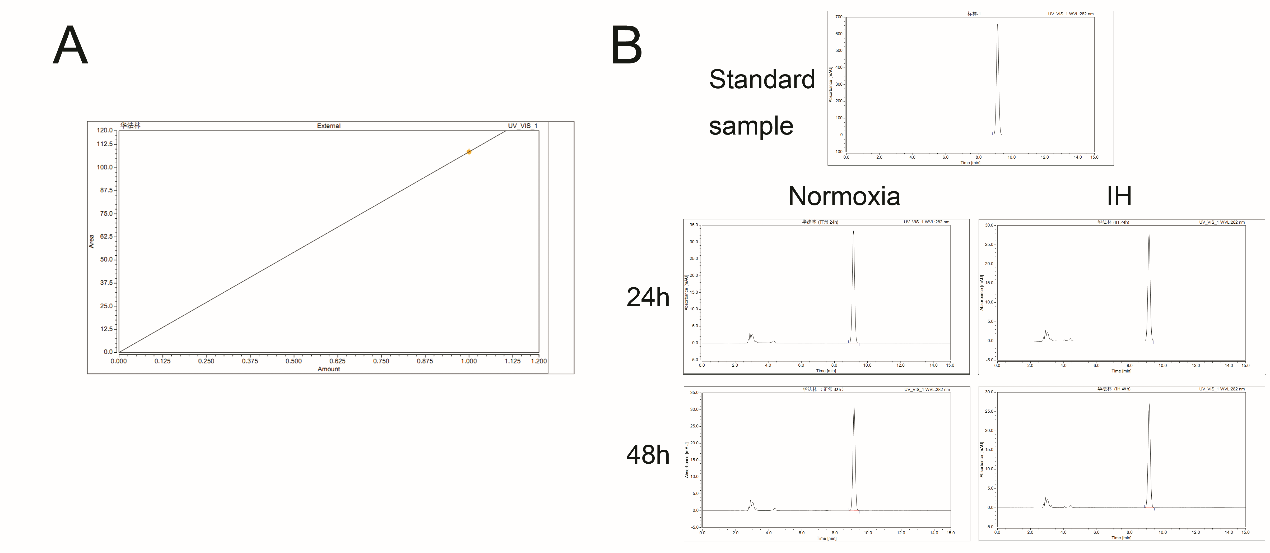


**A**: standard curve; **B**: chromatographs.

**eFigure 6 The body weight of rabbits at different time point**





There was no difference in body weight of rabbits between normoxia and intermittent hypoxia groups at all indicated time points.

**eFigure 7 The liver weight between normoxia and intermittent hypoxia groups**


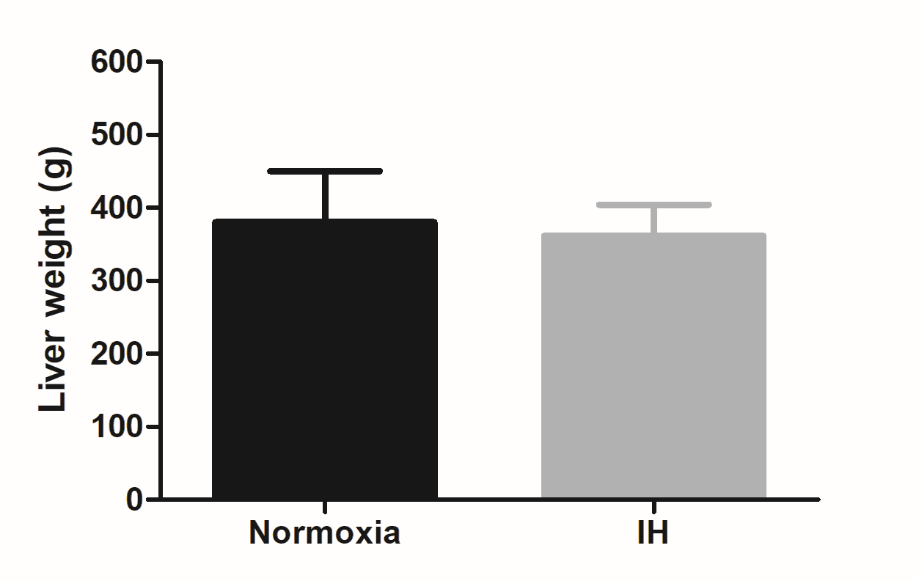


The mean liver weight of rabbits between groups was not different.
